# Prognostic value of the lymphocyte monocyte ratio in patients with colorectal cancer: A meta-analysis

**DOI:** 10.1097/MD.0000000000005540

**Published:** 2016-12-09

**Authors:** Wei Song, Kai Wang, Run-jin Zhang, Shu-bing Zou

**Affiliations:** Department of General Surgery, The Second Affiliated Hospital of Nanchang University, Nanchang, China.

**Keywords:** biomarker, colorectal cancer, lymphocyte to monocyte ratio (LMR), meta-analysis, prognosis

## Abstract

**Background:**

Inflammation plays a critical role in the pathogenesis and progression of cancer. A low lymphocyte-to-monocyte ratio (LMR) is reported be a poor prognostic factor in multiple malignancies. We performed a meta-analysis to evaluate the prognostic role of preoperative LMR in colorectal cancer (CRC).

**Methods:**

Studies investigating the prognostic role of preoperative LMR on survival in patients with CRC were systematically searched for in MEDLINE, EMBASE, Cochrane databases from inception up to August 2016. Pooled hazard ratios (HRs) for overall survival (OS), disease-free survival (DFS), and recurrence-free survival (RFS) were calculated using fixed-effects/random-effects models.

**Results:**

A total of nine studies comprising 8626 patients with CRC were included in the meta-analysis. The pooled analysis demonstrated that low LMR was significantly associated with decreased OS (HR: 0.63, 95% CI: 0.56–0.70, *P* < 0.001) and DFS/RFS (HR: 0.76, 95% CI: 0.68–0.84, *P* < 0.001). The negative prognostic impact of low LMR on OS was observed in patients with different ethnicity, treatment methods, cut-off values, and across disease stages.

**Conclusions:**

This meta-analysis demonstrates that low preoperative LMR is associated with worse survival in patients with CRC.

## Abstract 2

Supplemental Digital Content is available in the text

## 1 Introduction

Colorectal cancer (CRC) is the third leading cause of cancer-related death worldwide.^[1]^ Based on data from the American Cancer Society, it is estimated that approximately 142,820 new diagnosed cases and 50,830 deaths of cancer occur in the United States in 2013. Surgical resection is still the mainstay of treatment for patients with the non-metastatic disease, but unfortunately most of patients are not eligible for curative resection at the time of diagnosis.^[2]^ The 5-year survival rate for metastatic CRC remains poor.^[3]^ Therefore, it is necessary to detect prognostic markers for these patients to help individualize therapy and improve clinical outcomes.

It is well known that inflammation plays a critical role in the pathogenesis and progression of cancer.^[4]^ Inflammation indicators, such as the Glasgow Prognostic Score (mGPS), neutrophil to lymphocyte ratio (NLR), platelet to lymphocyte ratio (PLR), and C-reactive protein (CRP) have been reported to be useful prognostic markers in multiple cancers.^[5–8]^ Recently, the preoperative lymphocyte to monocyte ratio (LMR), which also reflects the degree of systemic inflammation, has been found to be linked to prognosis in patients with CRC.^[9–11]^ However, to the best of our knowledge, no meta-analysis assessing the correlation between preoperative LMR and the survival of CRC patients was performed. Thus, we conducted a meta-analysis to evaluate the effects of preoperative LMR on survival outcomes and the associations between LMR and the clinicopathological features in patients with CRC.

## 2 Materials and methods

### 2.1 Search strategies

We performed a comprehensive literature search of MEDLINE, EMBASE, Cochrane databases from inception up to August 2016. The following search terms were used in combination: “CRC” or “colorectal cancer” or “colorectal tumor” or “colorectal neoplasms” or “colon cancer” or “rectal cancer”, “LMR” or “lymphocyte-to-monocyte ratio” or “lymphocyte-monocyte ratio” or “lymphocyte to monocyte ratio” or “lymphocyte monocyte ratio”, “survival” or “prognostic” or “prognosis” or “clinical outcome”. Meanwhile, the references of eligible studies, relevant systematic reviews, and meta-analyses were also manually retrieved. This study was approved by The Institutional Review Board of the Second Affiliated Hospital of Nanchang University.

### 2.2 Eligibility criteria

Studies that met the following criteria were included: (1) CRC was pathologically confirmed; (2) investigating the prognostic role of preoperative LMR on overall survival (OS), disease-free survival (DFS), and/or recurrence-free survival (RFS); (3) studies supplied sufficient information for calculating hazard ratio (HR) and 95% confidence interval (CI); and (4) reporting the LMR cut-off value. Studies were excluded if they were: (1) reviews, comments, case reports, and conference abstract without original data; (2) overlapping or duplicate data; (3) non-English language studies.

### 2.3 Data extraction

The following information was captured using data abstraction forms: (1) study characteristics included first author's name, year of publication, country, ethnicity, survival analysis methods (multivariate, univariate), and time of follow-up. (2) Patient characteristics included age of patients, number of patients, disease stage (non-metastatic, metastatic, mixed: non-metastatic and metastatic), treatment, and cut-off value. (3) Outcome measures included HRs for OS, DFS, RFS as well as their 95% CIs, and clinicopathological features. HRs were extracted from multivariate or univariate analyses or estimated from Kaplan–Meier survival curves.^[12]^ Any conflicts were resolved by a third reviewer.

### 2.4 Quality assessment

The quality of each study was assessed according to the Newcastle-Ottawa Scale (NOS),^[13]^ which included an assessment of subject selection, comparability of groups, and clinical outcome. A total of nine items were extracted, and each item scored 1. The total scores ranged from 0 to 9. If scores are ≥7, the study is considered as high quality.

### 2.5 Statistical analysis

The meta-analysis was conducted by Review Manager 5.3 software (Cochrane Collaboration, Copenhagen, Denmark). The heterogeneity among eligible studies was quantified using the chi-squared based *Q*-statistic test. An *I*^2^ >50% and *P* < 0.10 was considered statistically significant. When there was no statistically significant heterogeneity, we used the fixed-effects model for pooling the results; otherwise, the random-effects model was applied. Survival outcomes were summarized as the logarithm of HR with 95% CIs by the generic inverse variance method. HRs and their 95% CIs were searched in the original articles or extrapolated using methods described by Tierney and Parmar.^[12,14]^ The associations between LMR and clinicopathologic features were expressed as risk ratios (RRs) and its 95% CIs. Subgroup analyses were conducted based on the patients’ ethnicity (Asian, Caucasian), disease stage (metastatic, non-metastatic, mixed), treatment method (surgery, chemotherapy, mixed), and the cut-off value of LMR (≥3, <3). Publication bias was estimated using funnel plot asymmetry tests.

## 3 Results

### 3.1 Search results

**Figure 1:**
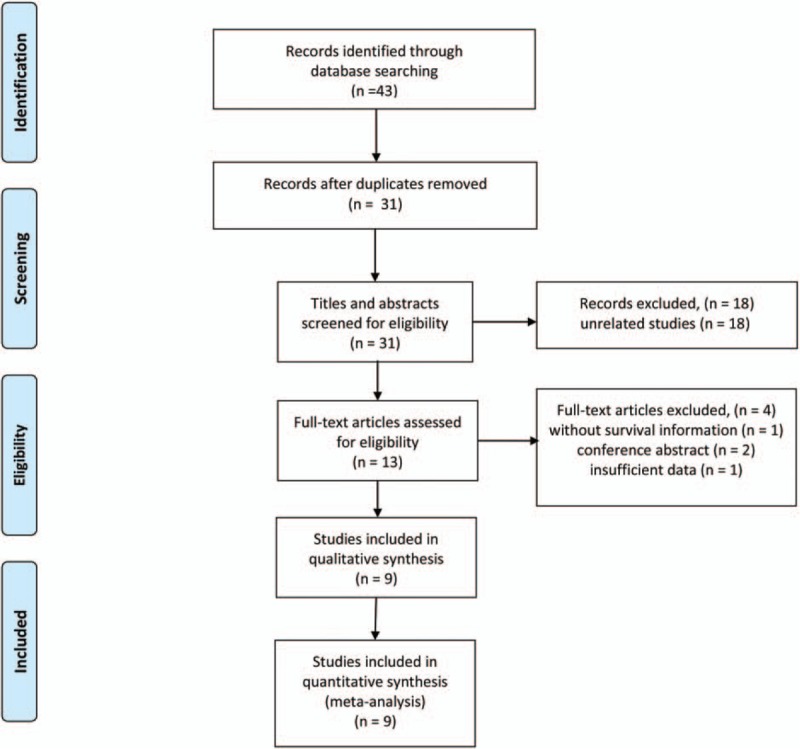
Flow diagram of the study selection process.

The literature search of electronic databases identified a total of 43 articles. After excluding duplicate articles, 31 potentially eligible studies were selected. Of these, 18 were excluded through titles and abstracts, leaving 13 articles for further evaluation. As a result, a total of nine studies comprising 8626 patients with CRC fulfilled all of the inclusion criteria.^[9,10,15–21]^ The PRISMA flow diagram of the study selection process was shown in Fig. 1.

Most of these studies have been published since 2015. The number of patients in each study ranged from 104 to 5336. Four studies were from China, 2 from Japan, 1 from USA, 1 from Austria, and 1 from South Korea. Seven studies investigated the prognostic value of LMR in OS, and 5 studies explored the prognostic impact of LMR in DFS/RFS. All included studies reported HRs and its 95% CI. The cut-off values for LMR ranged from 2.14 to 3.78, 5 studies used a LMR cut-off value ≥3, while 4 studies used a LMR <3. In methodological quality of studies, the NOS scores of all included studies were ≥7. Table 1 lists the detailed study characteristics.

**Table 1:**
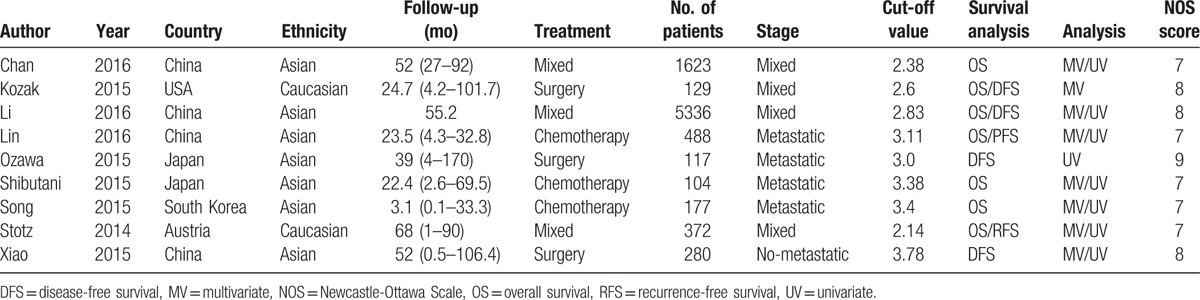
Characteristics of the studies included in the meta-analysis.

### 3.2 Meta-analysis

#### 3.2.1 Overall survival

**Figure 2:**
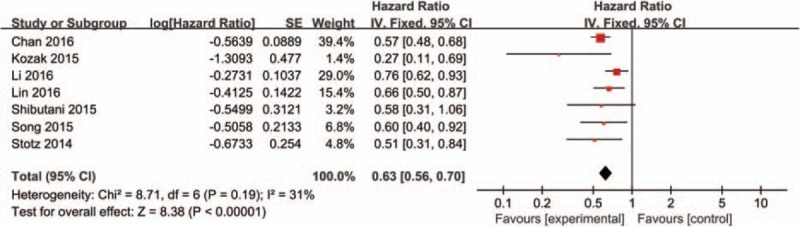
Forest plots for the association between LMR expression and OS. LMR = lymphocyte-to-monocyte ratio, OS = overall survival.

Seven studies comprising 8229 patients investigated the association between LMR and OS. The pooled analysis showed that low LMR had a significant association with decreased OS (HR: 0.63, 95% CI: 0.56–0.70, *P* < 0.001), with no heterogeneity between studies (*P* = 0.19, *I*^2^ = 31%) (Fig. 2).

Exploratory subgroup analyses stratified by disease stage, low LMR predicted decreased OS in patients with metastatic disease (HR: 0.63, 95% CI: 0.51–0.79, *P* < 0.001) and mixed subgroup including both non-metastatic and metastatic disease (HR: 0.59, 95% CI: 0.45–0.77, *P* < 0.001). Pooled HRs for OS according to the cut-off value, the OS rate was significantly worse in all subgroups. The highest negative effect of low LMR on OS was observed in patients with LMR < 3 (HR: 0.59, 95% CI: 0.45–0.77, *P* < 0.001). In addition, subgroup analyses suggested that low LMR predicted poor OS in patient with CRC, regardless of the ethnicity and treatment methods. Pooled HRs for OS according to subgroup analyses were shown in Table 2.

**Table 2:**
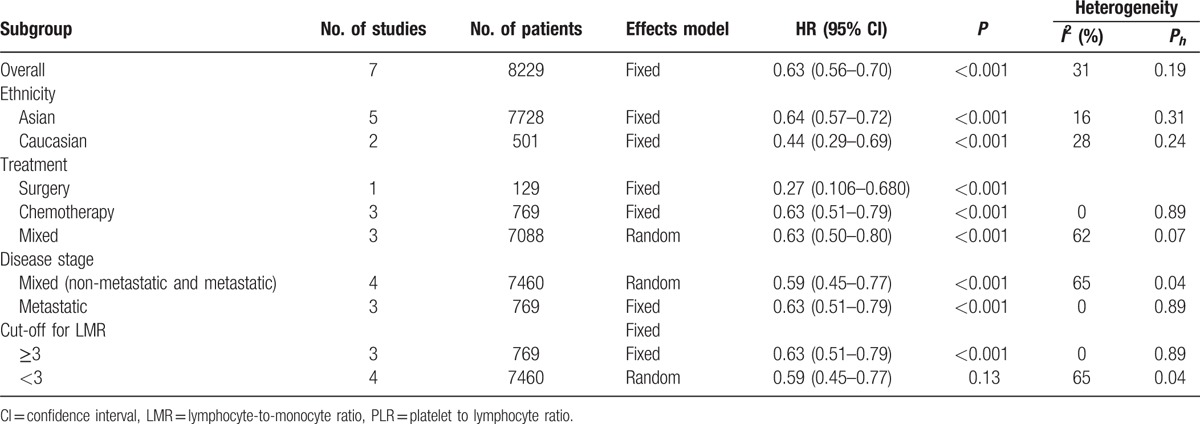
Pooled hazard ratios (HRs) for OS according to subgroup analyses.

### 3.3 Disease-free survival/recurrence-free survival

**Figure 3:**
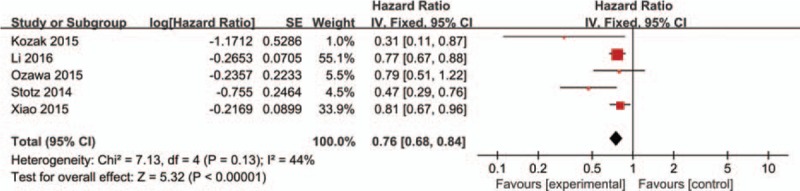
Forest plots for the association between LMR expression and DFS/RFS. LMR = lymphocyte-to-monocyte ratio, DFS = disease-free survival, RFS = recurrence-free survival.

Five studies involving 6234 patients evaluated the association between LMR and DFS/RFS. A combined analysis demonstrated that low LMR was significantly correlated with decreased DFS/RFS (HR: 0.76, 95% CI: 0.68–0.84, *P* < 0.001), with no heterogeneity between studies (*P* = 0.13, *I*^2^ = 44%) (Fig. 3).

### 3.4 Clinicopathological parameters

In the meta-analysis, we identified 3 clinical factors to explore the impact of LMR on the clinical features in CRC. Four studies reported on tumor differentiation. No significant difference was noted between the low LMR group and the high LMR group (RR: 0.82, 95% CI: 0.29–2.26, *P* = 0.69). Similarly, the results did not reveal a significant relationship between low LMR and T stage (RR: 1.09, 95% CI: 0.99–1.19, *P* = 0.08) and Lymph node metastasis (RR: 1.02, 95% CI: 0.93–1.12, *P* = 0.68).

**Figure 4:**
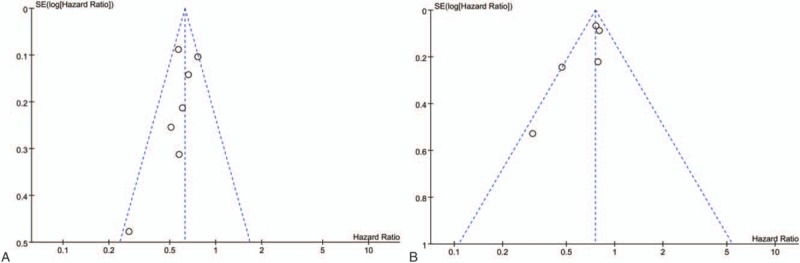
Forest plot of hazard ratios for OS (A) and DFS/RFS (B) in CRC. CRC = colorectal cancer, DFS = disease-free survival, OS = overall survival, RFS = recurrence-free survival.

Publication bias was evaluated using the Begg's funnel plot. The funnel plot of both OS and DFS was asymmetric, suggesting a high risk of publication bias (Fig. 4 A and B).

## 4 Discussion

In the present study, we identified 9 studies involving 8626 patients that investigate the prognostic role of preoperative LMR in patients with CRC. Our meta-analysis provides strong evidence that low LMR was significantly correlated with decreased OS and DFS/RFS. There was no significant heterogeneity among studies. Subgroup analyses were performed based on ethnicity, treatment methods, disease stages, and the LMR cut-off value. We stratified cut-off values into 2 subgroups: ≥3 and <3. Stratification by cut-off values and found that the OS rate was significantly worse in all subgroups. The highest negative effect of low LMR on OS was observed in patients with LMR <3, suggesting that lower LMR cut-off values may have more discriminative prognostic value for OS. The negative prognostic impact of low LMR on OS was observed in patients with different ethnicity, treatment methods, and across disease stages. Additionally, we further analyzed the correlations between preoperative LMR and clinicopathologic parameters. The results did not reveal a significant relationship of low LMR with tumor differentiation, T stage, and Lymph node metastasis.

The actual mechanisms of the prognostic impact of LMR in CRC are unclear. It has been suggested that cross-talk exists between the inflammatory response and tumor progression.^[4,22,23]^ Lymphocytes have a critical role in immunity by triggering antitumor immune responses. The lymphocyte count reflects the degree of responsiveness of the immune system of the host.^[24,25]^ Tumor-infiltrating lymphocytes (TILs) are important immune cells found within tumors and are responsible for antitumor immune responses.^[26]^ Amedei et al^[27]^ found that TILs cells from *Helicobacter pylori* infected patients with gastric cancer showed poor cytolytic activity while expressing helper activity for monocyte MMP-2, MMP-9, and VEGF production, which play an important role in angiogenesis, tumor invasion, and metastasis. Furthermore, low lymphocyte counts are thought to be responsible for an insufficient immunological response, which leads to inferior survival in multiple cancers.^[28,29]^

On the other hand, monocytes are also involved in tumor progression and metastasis.^[23]^ Tumor-associated macrophages (TMAs), which develop from circulating monocytes in the local tissues. TAMs can accelerate angiogenesis, invasion, migration, and tumor growth.^[30]^ The peripheral blood absolute monocyte count is considered to reflect the formation and/or presence of TAMs.^[20]^ Thus, a high monocyte count reflects an elevated tumor burden of cancer patients.

Given this background, the LMR reflects both the immune status of the host and the degree of tumor progression. A low LMR combined with the effects of low lymphocyte count and high monocyte count reflects insufficient antitumor immunity and a high tumor burden. Thus, LMR might be a stronger predictor of prognosis in patients with CRC.

Nevertheless, our study has several limitations. First, the cut-off value of LMR varied in each study. Second, publication bias was observed in both OS and DFS/RFS meta-analysis. The publication bias might be explained by several reasons. Studies with negative results are less likely to be published than those with positive results. Additionally, only published articles were included, and they were all written in English. Third, all included studies were retrospective analysis.

In conclusion, our study indicated that low preoperative LMR is confirmed to correlate with worse survival in patients with CRC, suggesting that LMR could provide essential information to inform prognosis and treatment decisions for CRC patients.

## Supplementary Material

Supplemental Digital Content
