# Supplementary material for: Prognostic value of the lymphocyte monocyte ratio in patients with colorectal cancer: A meta-analysis
Source: Medicine (Baltimore). 2016 Dec 9;95(49):e5540. doi: 10.1097/MD.0000000000005540 (PMC5266021; doi:10.1097/MD.0000000000005540)
Supplement: Supplemental Digital Content [file medi-95-e5540-s001.doc]

**Chochrane:**

**#1 (LMR or lymphocyte to monocyte ratio or lymphocyte monocyte ratio or lymphocyte-to-monocyte ratio or lymphocyte-monocyte ratio)**

**#2 MeSH descriptor Colonic Neoplasms explode all trees**

**#3 MeSH descriptor Rectal Neoplasms explode all trees**

**#4 MeSH descriptor Colorectal Neoplasms explode all trees**

**#5 (colon* cancer*) or (colon* carcinom*) or (colon* neoplas*) or (colon* tum*) or (rect* cancer*) or (rect* carcinom*) or (rect* neoplas*) or (rect* tum*) or (colorect* cancer*) or (colorect* carcinom*) or (colorect* neoplas*) or (colorect* tum*)**

**#6 (#2 OR #3 OR #4 OR #5)**

**#7 (survival or prognosis or recurrence or clinical outcome)**

**#8 #1 and #6 and #7**

**MEDLIME(Ovid SP):**

#**1 (LMR or lymphocyte to monocyte ratio or lymphocyte monocyte ratio or lymphocyte-to-monocyte ratio or lymphocyte-monocyte ratio).mp**

**#2 exp Colonic Neoplasms/**

**#3 exp Rectal Neoplasms/**

**#4 exp Colorectal Neoplasms/**

**#5 (colon* cancer* or colon* carcinom* or colon* neoplas* or colon* tum*).mp.**

**#6 (rect* cancer* or rect* carcinom* or rect* neoplas* or rect* tum*).mp.**

**#7 (colorect* cancer* or colorect* carcinom* or colorect* neoplas* or colorect* tum*).mp.**

**#8 2 or 3 or 4 or 5 or 6 or 7**

**#9 (survival or prognosis or recurrence or clinical outcome).mp**

**#10 1 and 8 and 9**

**Embase(Ovid SP):**

#**1 (LMR or lymphocyte to monocyte ratio or lymphocyte monocyte ratio or lymphocyte-to-monocyte ratio or lymphocyte-monocyte ratio).mp**

**#2 exp colon tumor/**

**#3 exp colon cancer/**

**#4 exp colon carcinoma/**

**#5 exp rectum cancer/**

**#6 exp rectum carcinoma/**

**#7 exp rectum tumor/**

**#8 exp colorectal cancer/**

**#9 exp colorectal carcinoma/**

**#10 exp colorectal tumor/**

**#11 (colon* tum* or colon* cancer* or colon* carcinom* or rect* cancer* or rect* carcinom* or rect* tum* or colorect* cancer* or colorect* carcinom* or colorect* tum*).mp.**

**#12 2 or 3 or 4 or 5 or 6 or 7 or 8 or 9 or 10 or 11**

**#13 (survival or prognosis or recurrence or clinical outcome).mp**

**#14 1 and 12 and 13**
